# Which recommendations are considered essential for outbreak preparedness by first responders?

**DOI:** 10.1186/s12879-017-2293-0

**Published:** 2017-03-07

**Authors:** Evelien Belfroid, Aura Timen, Jim E. van Steenbergen, Anita Huis, Marlies E. J. L. Hulscher

**Affiliations:** 1 Radboud University Medical Center, Radboud Institute for Health Sciences, IQ Healthcare, Geert Grooteplein 21, 6525 EZ Nijmegen, The Netherlands; 2 National Institute for Public Health and the Environment (RIVM), Centre for Infectious Disease Control, Preparedness and Response Unit, Antoni van Leeuwenhoeklaan 9, 3721 MA Bilthoven, The Netherlands

## Abstract

**Background:**

Preparedness is considered essential for healthcare organizations to respond effectively to outbreaks. In the current study we aim to capture the views of first responders on what they consider key recommendations for high quality preparedness. Furthermore, we identified the recommendations with the highest urgency from the perspective of first responders.

**Methods:**

We chose a multistep approach using a systematic Delphi procedure. Previously extracted recommendations from scientific literature were presented to a national and two international expert panels. We asked the experts to score the recommendations based on relevance for high quality preparedness. In addition we asked them to choose the ten most urgent recommendations.

**Results:**

Starting with 80 recommendations from scientific literature, 49 key recommendations were selected by both international expert panels. Differences between both panels were mainly on triage protocols. In addition, large differences were found in the selection of the ten most urgent recommendations.

**Conclusions:**

In this study infectious disease experts selected a set of key recommendations representing high quality preparedness and specified which ones should be given the highest urgency when preparing for a future crisis. These key recommendations can be used to shape their preparedness activities.

## Background

Since 1940, the frequency of outbreaks and the diversity of infectious disease pathogens have increased significantly [1, 2]. Most outbreaks are successfully dealt with at the local or regional level, but some have the propensity to become epidemics. Outbreaks trigger anxiety in the general population and require prompt and adequate actions from healthcare organizations and healthcare professionals [3]. Healthcare organizations have to deal with an increased number of (potentially) infected patients and have to accommodate new procedures and algorithms that interfere with daily routines. Moreover, professionals with various backgrounds of expertise need to work together in a coordinated way to respond to the outbreak [4]. Unfortunately, recent outbreak evaluations show that outbreak response is often suboptimal; there is considerable room for improvement [5, 6].

For healthcare organizations preparedness is considered essential to respond quickly and effectively to outbreaks in order to minimize the spread of pathogens and to reduce the number of infected persons. Preparedness requires an operational mindset focused on the development of formal procedures and guidelines in all preparedness phases, even during the period when there is no threat or outbreak. Many studies conclude that while at the national level preparedness guidelines and procedures are often in place, regional healthcare organizations are not always fully prepared [7–9]. It is a challenge for first responders to optimally prepare for outbreaks. The process of outbreak preparedness is time consuming and costly for healthcare organizations and asks for a shared responsibility of various actors in the field [10]. Therefore it is important that preparedness activities are efficient and effective, i.e. actually contribute to a better response. There are, however, no widely accepted standards for optimal outbreak preparedness for first responders as -due to the infrequency and the acute nature of outbreaks- there is little systematic evidence linking preparedness activities to response outcomes [10, 11].

This lack of clear and agreed upon standards does not guide first responders in optimal preparedness. The CDC [12], ECDC [13] and RAND corporation [14] developed tools to support preparedness planners and assess the level of preparedness. These tools, however, are intended primarily for public health organizations and are not specifically aimed at first responders, or focus solely on quality improvement without quality measurement, or focus on an influenza pandemic only.

Previously, we systematically reviewed the scientific preparedness literature and generated a series of 80 generic recommendations representing optimal outbreak preparedness for first responders originating from industrialized countries (low and middle income country settings were excluded) (Huis, A., Belfroid E., Klein Breteler, J., van Steenbergen, J., Hulscher, M. Defining and improving healthcare system's preparedness for infectious disease outbreaks: a systematic review identifying generic key recommendations and their connections to continuous quality improvement. Submitted). First responders were defined as organizations which are directly involved in providing healthcare during an infectious disease outbreak (including primary-, secondary-, tertiary- and home and community care providers), or indirectly involved in providing healthcare services during an outbreak (including local health departments, and clinical diagnostic laboratories). In the current study we aim to capture the views of first responders on the relevance of each recommendation as a criterion for high quality preparedness. Furthermore, we aim to identify those recommendations that have the highest urgency to be implemented from the perspective of the first responders. We compare two expert groups, the ECDC National Focal Points for preparedness and response, and authors of important papers on outbreak preparedness.

## Methods

**Fig. 1:**
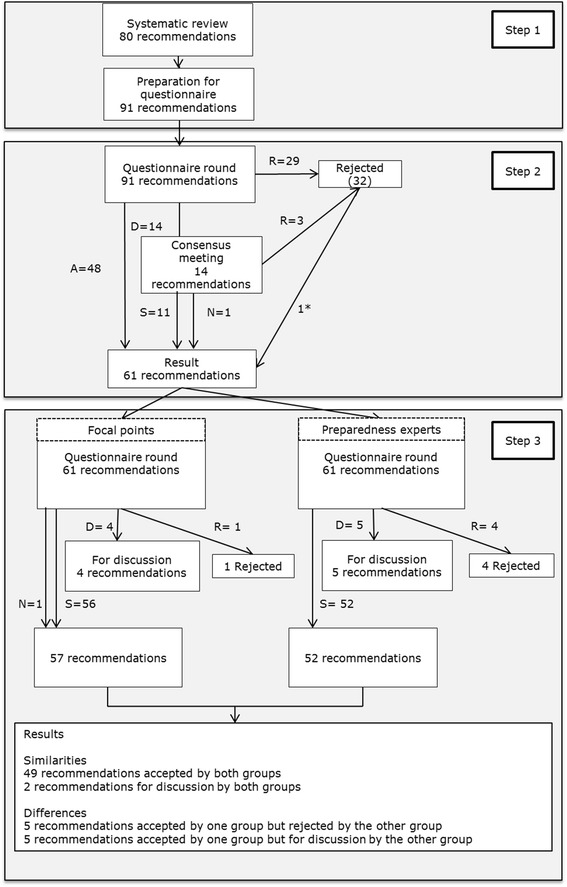
Selection procedure key recommendations. Legenda: S = Selected, D = Discussion, R = Rejected, N = New added. *This recommendation was rejected by the national experts but added to step 3 because in the Dutch legislation compulsory vaccination is forbidden

We chose a multistep approach using a Delphi procedure [15] to select a set of key recommendations representing high quality infectious disease preparedness from a first responder’s perspective. Recommendations extracted from scientific literature (Huis, A., Belfroid E., Klein Breteler, J., van Steenbergen, J., Hulscher, M. Defining and improving healthcare system's preparedness for infectious disease outbreaks: a systematic review identifying generic key recommendations and their connections to continuous quality improvement. Submitted) were prepared for the questionnaire round (step 1), see Fig. 1. The recommendations were presented to a national panel (step 2) in order to pilot the questionnaire, clarify the wording, and condense the number of recommendations resulting in an amended questionnaire. The amended questionnaire was presented to two international expert panels (step 3), the ECDC National Focal Points for preparedness and response, and authors of important papers on outbreak preparedness. The results from these two international expert panels were compared to gain insight into their perspectives. Formal ethical approval from a medical ethical committee was not required for this research in the Netherlands since it does not entail subjecting participants to medical treatment or imposing specific rules of conduct on participants. All the experts consented to participate in the study and were aware that their responses would be used for research purposes.

### Step 1: From systematic review to questionnaire

The systematic review yielded 80 recommendations (Huis, A., Belfroid E., Klein Breteler, J., van Steenbergen, J., Hulscher, M. Defining and improving healthcare system's preparedness for infectious disease outbreaks: a systematic review identifying generic key recommendations and their connections to continuous quality improvement. Submitted). The ones with a more general formulation which included more than one concept were split into concept specific recommendations. The recommendations were processed in an online questionnaire (using Limesurvey, an open source web application to develop, publish and collect responses to online surveys) to be administered to the panels in the next steps.

### Step 2: National expert panel

#### Expert panel

For the national panel a multidisciplinary group of experts on outbreak preparedness were invited. The main selection criteria for inclusion in the panel were the variety of specialties actually involved in outbreak preparedness and diversity of practice settings. The panel was invited per e-mail or telephone in November 2014.

#### Assessment of key recommendations

We asked the experts to appraise the relevance of the recommendations using a nine point Likert scale (1 = highly irrelevant, 9 = highly relevant). Each group of recommendations also contained an open textbox that the experts could use for remarks or to add a recommendation.

Data were analyzed using SPSS, median relevance scores were calculated for each recommendation. Recommendations with a median score > 7 and agreement (>70% of the scores in the highest tertile) were directly selected. Recommendations with a median score >7 and no agreement (<70% of the scores in the highest tertile) were submitted for discussion. The remarks of the experts on recommendations with a median score of seven were assessed by the researchers (EB, AH, JvS, AT, MH). If the remarks suggested a textual amendment of the recommendation the recommendation was also marked “discussion”. Recommendation with a median score < 7 were rejected.

#### Face-to-face meeting

In this meeting, recommendations previously marked for discussion could be accepted without change, altered textually or rejected by group discussion. The face-to-face meeting was held on December 15th 2014. The online questionnaire was revised on the basis of the results from step 2 and served as input for step 3.

### Step 3: Two international expert panels

To reach first responders in a high number of countries with substantial interregional differences we decided to approach the National Focal Points for Preparedness and Response and urged them to fill out the questionnaire from a first responder’s perspective in their country.

#### Expert panels

The first panel consisted of the ECDC National Focal Points for preparedness and response or their alternates designated by the EU member states to represent them in ECDC meetings. We aimed for one response per country from the National Focal Point or the alternate National Focal Point. The panel was invited per e-mail to participate. The panel was conducted between April 2015 and July 2015. The second panel consisted of international experts on outbreak preparedness who were approached per email or letter. We invited the first, second and last authors of the papers included in the previously conducted systematic review because of their scientific reputation in the field illustrated by peer-reviewed publications on the subject of front line preparedness(Huis, A., Belfroid E., Klein Breteler, J., van Steenbergen, J.,Hulscher, M., Defining and improving healthcare system's preparedness for infectious disease outbreaks: a systematic review identifying generic key recommendations and their connections to continuous quality improvement. Submitted). The panel was invited between July 2015 and February 2016. Non-responders from both groups received at least one reminder per letter or e-mail. We aimed for 7–15 participants per expert group as this is the recommended number of participants [15].

In step 3, we submitted the revised questionnaire for assessment to both international panels.

#### Assessment of key recommendations

The assessment procedure of recommendations was the same as previously explained in step 2. In addition, we asked the experts to “select the ten recommendations out of the total number of 61 you consider the ones with the highest urgency to implement”. Recommendations were considered urgent if more than 30% of the experts chose them as such.

## Results

### Step 1

The 80 recommendations presented in the systematic review were systematically translated into 91 recommendations for the questionnaire. For example: ‘Healthcare institutions should provide ongoing education programs about infection prevention for employees, and they should organize regular disaster drills and exercises’ was translated into two recommendations: ‘Healthcare organizations should provide ongoing infection prevention education programs for employees’ and ‘Healthcare organizations should organize regular infectious disease drills/exercises’. Six domains emerged from the raw data and the recommendations were categorized accordingly:

- *‘construction and maintenance of the outbreak preparedness plan’* which describes the development and updating of the preparedness plan in collaboration with relevant partners,
- *‘support for health professionals, patients and families’* which describes education and training, infection control measures and psychosocial assistance for healthcare professionals,
- *‘surge capacity’* which describes triage, infrastructure and equipment,
- *‘communication to the public, patients and families’* which describes communication strategies,
- *‘coordination and collaboration’* which describes coordination and collaboration with the relevant stakeholders, and
- *‘facilitators for implementation of plans’* which describes facilitating recommendations for the implementation of plans and protocols.

### Step 2

#### Expert panel

Nineteen Dutch experts were invited to fill out the digital questionnaire. Fourteen experts filled out the questionnaire (response rate 74%). The expert group consisted of the following experts; infectious disease specialist, infection control preventionist, General Practitioner, medical microbiologist, public health specialist, public health nurse, disaster management expert, and a virologist). Reasons for non-response were: a lack of expertise on the subject (*n* = 2), personal circumstances (*n* = 2) and unknown (*n* = 1).

#### Assessment of key recommendations

Forty-eight recommendations were directly selected. Fourteen recommendations were marked ‘for discussion’ (12 recommendations had a median of eight but less than 70% of the scores in the highest tertile and two had a median of seven and the experts’ comments suggested a textual amendment of the recommendation). Twenty-nine recommendations were rejected. The rejected recommendations described, to name some examples, addressing psychosocial needs of healthcare workers, vaccination of healthcare personnel and prioritization of support services.

#### Face-to-face meeting

All experts that filled out the digital questionnaire (*n* = 14) were invited for the meeting. Six of the 14 experts (43%) attended the meeting. The 14 recommendations with the label “discussion” and the two ones with textual amendments were reviewed. This resulted in two recommendations being accepted, nine recommendations being textually amended, one newly added recommendation, and three recommendations being rejected.

The 60 accepted recommendations in step 2 were included in step 3. One recommendation that was rejected in step 2 was included in the questionnaire for step 3 because in the Dutch legislation compulsory vaccination is forbidden (see Table 2, recommendation number 12). In total 61 recommendations were included in the questionnaire, see Table 2.

### Step 3

#### Expert panels

##### National Focal Points

Thirty countries were invited to fill out the digital questionnaire. We sent the invitation e-mail to 30 ECDC National Focal Points for Preparedness and Response and 27 alternates (three countries did not have an alternate). Fifteen experts filled out the questionnaire from 14 different countries (country response rate 47%): Italy, Germany, Norway, Lithuania, Denmark, Slovenia, Malta, Ireland, Belgium, the Netherlands, Cyprus, Romania, Hungary and Croatia (Table 1). One of these responses was incomplete. Reasons for non-response were unknown (*n* = 16 countries). From one country we received two responses, one from the National Focal Point and one from the alternate National Focal Point. Because this was the only country with more than one response, we decided to exclude the latest arriving response. Including both responses in the analysis would give this country more weight than others in the results (each response is valued equal). We performed the analysis on 14 responses (of which one was incomplete, the respondent assessed the first ten recommendations) from 14 countries.

**Table 1:** Respondent characteristics

	National Focal Points	Preparedness experts
Response	N total = 14 N complete = 13 N incomplete = 1	N total = 8 N complete = 7 N incomplete = 1
Countries	Belgium Croatia Cyprus Denmark Germany Hungary Ireland Italy Lithuania Malta Netherlands Norway Romania Slovenia	Australia (2) Israel (2) Italy (2) USA (2)
Years of experience	Mean: 7.93 SD: 6.28	Mean: 15.38 SD: 5.66
Type of organization	(National) Institute for Public Health (10) Ministry of health (4)	University (4) National Institute for Infectious Diseases/Public Health (2) Ministry of Health + University Academic hospital

##### Preparedness experts

While applying the selection criteria 60 experts were identified and invited to fill out the questionnaire. Three of the selected experts could not be invited due to unavailable contact information. Eight experts filled out the questionnaire (response rate 14%), see Table 1. One of the responses was incomplete. Reasons for non-response were: contact information incorrect (*n* = 6), no expert on the subject (*n* = 1) and unknown (*n* = 42). Our experts ranged from clinical to emergency management and public health. Five of the preparedness experts worked at a university. The expert group consisted of two professors of public health, a director of general intensive care unit, an epidemiologist, a professor of pediatrics (head of infectious diseases unit), an infectious diseases consultant, a professor of environmental and occupational health (Institute for Biosecurity), a senior consultant on emergency management who is also a faculty member in the emergency medicine department at a university. The preparedness experts had between 10 and 26 years of experience in their current function.

#### Assessment of key recommendations

Fifty-six recommendations were selected by the National Focal Points, see Table 2. Four recommendations were marked ‘for discussion’. One recommendation was rejected and one recommendation was newly added. Fifty-two recommendations were selected by the preparedness experts, see Table 2. Five recommendations were marked for ‘discussion’. Four recommendations were rejected and no new recommendations were added.

**Table 2:** Key recommendations

Domain	No	Key recommendation	National Focal Points	Prep. experts
Construction and maintenance of the outbreak preparedness plan	1	Staff responsible for outbreak preparedness planning from small healthcare organizations should have access to education and training in outbreak planning.	S	S
	2	The organization's infectious disease preparedness plan should be updated.	S	S
	3	Healthcare organizations should develop their organizational infectious disease preparedness plan in a multidisciplinary internal committee.	S MU	S
	4	Healthcare organizations should tune their organizational infectious disease preparedness plan with all (local/regional/national) organizations that they interact with during outbreaks.	S MU	S MU
	5	The organization's infectious disease preparedness plan should be generic (flexible and adaptable to the actual situation).	S MU	S MU
	6	The organization's infectious disease preparedness outbreak plan should correspond with the national guidelines, but should deviate to fit the local situation.	S MU	S
	7	Resources for developing, testing, and updating a preparedness plan should be made available.	S MU	S
	8	Healthcare organizations should have a dedicated staff position responsible for infectious disease preparedness.	D	D
	9	The organization’s outbreak preparedness plan and its updates should be disseminated and implemented in multiple and various ways by the responsible management.	S MU	S
	10	Healthcare workers should be able to access the organizations preparedness plan (for example on the intranet).	S	S
Support for health professionals, patients and families	11	An infectious disease preparedness plan should include items for staff protection; nb in case of uncertainty protection should start at the highest required level in the actual setting whereby the protection of employees is guaranteed, adapted for the specific situation.	S MU	S MU
	12	A procedure should be developed to mandate the designated employees to receive the (for the outbreak designated) vaccine and/or antiviral prophylaxis^a^.	S	S
	13	Drills and exercises to assess how various healthcare facility's plans interact should be multidisciplinary.	S	S
	14	Healthcare organizations should evaluate their level of preparedness.	S	S MU
	15	Healthcare organizations should provide ongoing infection prevention education programs for employees.	S	S
	16	Healthcare organizations should organize regular infectious disease drills/exercises	S MU	S
	17	Healthcare workers should be educated and trained in outbreak handling.	S	S
	18	Training materials for infection control measures should be available at the healthcare organization.	S	S
	19	Instructors providing training should be trained instructors.	S	D
	20	The senior management should identify staff that has to participate in training programs.	S	S
	21	The senior management should allocate staff to the defined roles.	S	D
	22	The senior management should verify that the identified staff participated in the training.	S	S
	23	All designated professionals should be trained.	S MU	S MU
	24	Healthcare organizations should anticipate addressing mistrust, fear, moral maintenance, and sustainability of health care workers.	S	S
	25	All relevant employees should be fit-tested for PPE use.	S	D
	26	The organization should appoint a person who is available for questions from the staff on PPE use.	S	S
	27	Compliance of healthcare workers with transmission-based precautions should be enforced by diverse strategies.	S	R
	28	An infectious disease preparedness plan should include items supporting infection control (including measures to prevent contamination) for all phases of the outbreak.	S MU	S MU
	29	Healthcare organizations should take into account that they might need to put potentially infectious (asymptomatic) healthcare workers on administrative leave.	S	S
	30	Healthcare organizations should plan to monitor the health of exposed healthcare workers for the maximum incubation period.	S	S
	31	Healthcare organizations should plan to screen patients with infectious disease symptoms.	S	S
	32	Specific protocols for high risk procedures should be available for high risk infectious diseases.	S	S
Surge capacity	33	Health care organizations providing patient care should have an overview of the general features of the organization (e.g. logistic structures including private rooms, toilets, wards, rooms dedicated to infectious disease, rooms equipped with negative pressure systems, safe waste disposal).	S	S
	34	Health care organizations should plan to expand their capacity.	S	S MU
	35	Hospitals should have at least one isolation room which is technically well-equipped and logistically adequate.	S	S
	36	Healthcare organizations should have access to adequate laboratory facilities for screening and follow-up.	S	S
	37	Healthcare organizations should have access to a single, simple reporting framework to minimize the administrative burden to reporting.	D	D
	38	Healthcare organizations should have a plan to access, coordinate, and increase labor resources for continued and expanded care.	S	S
	39	Healthcare organizations should have an up to date inventory of the total number of personnel (medical and non-medical staff) with patient contact and without patient contact.	S	S
	40	Healthcare organizations should have an access plan in place for stockpiling and distribution.	D	S MU
	41	Senior management should prepare to provide adequate resources to respond to an outbreak.	S MU	S
	42	Healthcare organizations should prepare for immediate installation of a surveillance network to monitor the burden of disease during an outbreak in relation to the capacity of healthcare.	S	S
	43	Healthcare organizations should prepare for monitoring of exposed cases.	S	S
	44	Healthcare organizations should plan to inform ambulance and organization’s (hospital) staff when a case was transferred.	S	S
	45	Healthcare organizations should have triage protocols for infectious disease outbreaks.	S	S
	46	Triage protocols should be developed supra institutional.	S	R
	47	Triage protocols should include an ethical framework to manage competing priorities to relevant pathways of decision making.	S	R
	48	Healthcare organizations should have a triage system for infectious disease outbreaks.	S	S
	49	A preparedness plan should include objective criteria to activate and stop triage protocols.	D	S
	50	Triage protocols should be tuned regionally.	S	R
	51	The public health preparedness plan should define identify and designate staff for supporting and maintaining (suspected/probable/possible/confirmed) cases in home isolation.	S	S
Communication to the public, patients and families	52	Healthcare organizations should have communication strategies for patients and their families.	S	S
Coordination and collaboration	53	Organizations should have a multidisciplinary organizational preparedness committee to ensure the organization is well prepared.	S	S
	54	Ensure that an outbreak coordinator can be appointed in an outbreak situation.	S	S
	55	Healthcare organizations should prepare for installing an outbreak control group (e.g. an outbreak management team) in case of a threat to coordinate the response.	S MU	S
	56	A communication and coordination system between each healthcare organization and the local/regional/state/country public health authorities should be established.	S MU	S
	57	Healthcare organizations should collaborate, coordinate, and communicate with key regional stakeholders for outbreak preparedness.	S	S MU
	58	Healthcare organizations should plan to establish a regional outbreak control group to exercise authority and direction over resources in the region.	R	S
	59	Healthcare organizations should develop an internal information channel to ensure up-to-date information is factual, accurate, and reliable while preventing information overload.	S MU	S
Facilitators for implementation of plans	60	Healthcare organizations should have strategies to ensure effective and responsive leadership.	S	S
	61	Senior-level management should be prepared to provide good leadership.	S	S
New recommendations added		Healthcare organizations should do a risk assessment and develop scenarios for the most probable outbreak situations.	Added by the National Focal Points	

Legenda: *S* selected, *D* discussion, *R* rejected, *MU* selected for most urgent

^a^This recommendation was rejected by the national experts in step 2 but the authors added the recommendation to step 3 because in the Dutch legislation compulsory vaccination is forbidden

There was a large amount of overlap between both groups. Fourty-nine recommendations were accepted by both the National Focal Points and the preparedness experts and two recommendations were marked ‘for discussion’ by both groups. Ten recommendations were appraised differently by the two panels. Five recommendations were selected by one group but rejected by the other group and five recommendations were accepted by one group but ‘for discussion’ by the other group. Most of the differences were in the domain ‘surge capacity’ and described the triage protocols.

#### Ten most urgent recommendations selected by National Focal Points and preparedness experts

Table 3 displays the selected recommendations by both groups. Five recommendations were selected by both groups. Four of these recommendations concerned the development of the preparedness plan and one recommendation the training of healthcare professionals. In addition, the National Focal Points selected nine and the preparedness experts four different recommendations as ‘most urgent’. Out of the nine additionally selected recommendations by the National Focal Points four described the preparedness plan, one preparedness exercises, two described sharing information with other organizations, one described the provision of resources and one the installation of an outbreak control group. Out of the four additionally selected recommendations by the preparedness experts one described an access plan for stockpiling and distribution, one the evaluation of the level of preparedness, one the plan to expand the healthcare organization’s capacity and one the collaboration with regional key stakeholders.

**Table 3:** Selected most urgent recommendations

	Selected 10 most urgent recommendations National Focal Points (*N* = 13)	Selected 10 most urgent recommendations preparedness experts (*N* = 7)
Agreements	The organization's infectious disease preparedness plan should be generic (flexible and adaptable to the actual situation) (*N* = 10, 77%) Healthcare organizations should tune their organizational infectious disease preparedness plan with all (local/regional/national) organizations that they interact with during outbreaks (*N* = 5, 38%) An infectious disease preparedness plan should include items for staff protection; nb in case of uncertainty protection should start at the highest required level in the actual setting whereby the protection of employees is guaranteed, adapted for the specific situation (*N* = 5, 38%) An infectious disease preparedness plan should include items supporting infection control (including measures to prevent contamination) for all phases of the outbreak (*N* = 4, 31%) All designated professionals should be trained (*N* = 4, 31%)	The organization's infectious disease preparedness plan should be generic (flexible and adaptable to the actual situation) (*N* = 3, 43%) Healthcare organizations should tune their organizational infectious disease preparedness plan with all (local/regional/national) organizations that they interact with during outbreaks (*N* = 4, 57%) An infectious disease preparedness plan should include items for staff protection; nb in case of uncertainty protection should start at the highest required level in the actual setting whereby the protection of employees is guaranteed, adapted for the specific situation (*N* = 3, 43%) An infectious disease preparedness plan should include items supporting infection control (including measures to prevent contamination) for all phases of the outbreak (*N* = 5, 71%) All designated professionals should be trained (*N* = 3, 43%)
Differences	Healthcare organizations should develop their organizational infectious disease preparedness plan in a multidisciplinary internal committee (*N* = 7, 54%) A communication and coordination system between each healthcare organization and the local/regional/state/country public health authorities should be established (*N* = 6, 46%) Resources for developing, testing, and updating a preparedness plan should be made available (*N* = 5, 38%) Healthcare organizations should organize regular infectious disease drills/exercises (*N* = 5, 38%) Healthcare organizations should develop an internal information channel to ensure up-to-date information is factual, accurate, and reliable while preventing information overload (*N* = 5, 38%) The organization's infectious disease preparedness outbreak plan should correspond with the national guidelines, but should deviate to fit the local situation (*N* = 4, 31%) The organization’s outbreak preparedness plan and its updates should be disseminated and implemented in multiple and various ways by the responsible management (*N* = 4, 31%) Senior management should prepare to provide adequate resources to respond to an outbreak (*N* = 4, 31%) Healthcare organizations should prepare for installing an outbreak control group (e.g. an outbreak management team) in case of a threat to coordinate the response (*N* = 4, 31%)	Healthcare organizations should have an access plan in place for stockpiling and distribution (*N* = 4, 57%) Healthcare organizations should evaluate their level of preparedness (*N* = 3, 43%) Health care organizations should plan to expand their capacity (*N* = 3, 43%) Healthcare organizations should collaborate, coordinate, and communicate with key regional stakeholders for outbreak preparedness (*N* = 3, 43%)

## Discussion

In this study infectious disease experts selected a set of key recommendations representing high quality preparedness and specified which ones should be given the highest urgency when preparing for a future crisis.

Several attempts have been made to develop recommendations for outbreak preparedness [11–14]. These recommendations differ in perspective, number of healthcare organizations the recommendations apply to, type of healthcare organization and, level of detail and specificity. Our key recommendations, in contrast to the existing recommendations, are specifically developed from the perspective of first responders to guide them in selecting relevant preparedness activities for their organization, and not from a regional or national perspective. When aggregating this information at regional level, policy makers can map the strengths and weaknesses of the region as a whole and decide upon specific interventions to improve overall preparedness.

Several patterns emerge when analyzing the final set. Firstly, the selected recommendations do not focus on psychosocial aspects of outbreak preparedness, such as ‘childcare for employees’ and ‘mental support for employees’. Recommendations regarding psychosocial aspects have been rejected in step 2. The international expert panels in step 3 had the option to add new recommendations but no recommendation regarding psychosocial aspects was added to the list. During large outbreaks, these aspects are highly important to ensure employees work attendance and commitment to sustained efforts, while possibly finding themselves at risk of acquiring and transmitting the disease to their family and friends [16]. We assume these recommendations were rejected because the expert panel mainly consisted of doctors and policy makers who tend to focus on the organizational and medical aspects of preparedness, rather than the conditions that facilitate health care professionals to attend work. Literature shows that psychosocial needs need to be taken into account [17–20]. More research is needed to determine the specific needs of healthcare workers during outbreaks and the corresponding preparedness activities. Secondly, we found a high level of consistency (49 recommendations) in the selection of relevant commendations from the initial set, by both panels. This shows that almost all preselected recommendations were considered important by our experts teams. Thirdly, both panels endorsed the importance of the preparedness for triage, a key component of outbreak response. Both groups agreed that healthcare organizations need to have triage protocols. The way to prepare for triage was, however, rather different for both panels. While the National Focal Points considered the supra institutional development of triage protocols, the inclusion of an ethical framework in the protocol and regional tuning of the protocols relevant key recommendations, the preparedness experts rejected them. Contrarily, the key recommendation on objective criteria to activate and stop triage protocols was rejected by the National Focal Points but accepted by the preparedness experts. This reflects a longstanding debate on triage protocols. It is practically impossible to predict what situations might develop in an outbreak so it can be rather challenging to develop universal triage protocols beforehand. On the other hand, triage is a very political sensitive topic. It can thus be useful to consider a variety of aspects in detail beforehand and incorporate them in a triage protocol. There is no literature available that provides a sufficient evidence base to develop key recommendations regarding triage [21].

Considering the ‘most urgent recommendations’ there are five recommendations that are selected by both expert groups. This is a strong signal that those five recommendations have a high urgency. Organizations have to develop a generic plan, which is flexible to allow for changes to reflect new developments during a specific outbreak. Furthermore, they should work together and tune their respective plans. Also the staff protection and infection control measures are considered urgent by both groups. In addition training of all designated professionals is considered urgent. It is not surprising these five recommendations were selected. The preparedness plan, training and exercises and infection control measures receive a great deal of attention in the preparedness literature, implying there is a general consensus that these elements are important (Huis, A., Belfroid E., Klein Breteler, J., van Steenbergen, J.,Hulscher, M. Defining and improving healthcare system's preparedness for infectious disease outbreaks: a systematic review identifying generic key recommendations and their connections to continuous quality improvement. Submitted).

In addition, we found several differences in the ‘most urgent recommendations’ selected meaning that not all recommendations are equally important for the different groups. Differences between both groups in the selected’10 most urgent’ can presumably be explained by the different perspectives of both respondent groups. The National Focal Points selected several recommendations about the development of the preparedness plan and other formal procedures. The preparedness experts selected the more practical recommendations. When improving outbreak preparedness it is impossible to focus on the total set of recommendations because of the size of the set, so it makes sense to concentrate on the most urgent recommendations. The differences between both groups show us that the background and perspective of the professional is very influential in the choice for the most urgent recommendations. Therefor it is very important that all relevant stakeholders from the region are included in outbreak preparation so that no perspectives are left out.

Our study has several limitations. Firstly, we realize that our data concern only rich industrialized countries and the recommendations therefore are not easily applicable to low income countries with completely different context, infrastructure, priorities and resources. However, we concentrated on the wealthy counties as they have the possibilities, research, and experts to achieve the highest preparedness standards. A similar systematic approach should be used (systematic review and expert consensus procedure) to select recommendations for low- and middle-income countries. Secondly, the aim of the study was to develop a set of recommendations for the first local response in an outbreak situation. Although we urged the international experts to fill out the questionnaire from that perspective, we realize that these might be biased to more managerial aspects. However, we observed high similarity between the selected items by the “true first responders” in the Dutch panel with those of the international experts of “quasi first-responders”. Another limitation is the focus on outbreaks and not all hazard preparedness. We did so because outbreaks require unique recommendations because of the transferable potential of pathogens and an all hazard approach would miss, for outbreaks specific, relevant recommendations. One of the strengths of our study is the inclusion of a wide variety of experts in the field of outbreak preparedness. All our respondent groups (*n* = 14, *n* = 14 and *n* = 8) contained a sufficient number of participants as compared to the recommended 7–15 number of participants [15]. The national panel consisted of a wide variety of disciplines involved in outbreak preparedness. The panel of the National Focal Points on preparedness and response covered the entire European Union and the respondent panel of the preparedness experts had a worldwide coverage including different specialties and experienced respondents.

## Conclusions

We present a set of generic recommendations, including a prioritization of the most urgent ones. When preparing for the next crises, these can provide the basis for frontline organizations to guide decisions on how and where to start, as well as to identify weaknesses. Outbreak preparedness requires a solid scientific base. While this field of research is rather new, more efforts are needed to provide systematic evidence on high quality preparedness, ideally linking it to outbreak response outcomes.
